# CGGBP1 mitigates cytosine methylation at repetitive DNA sequences

**DOI:** 10.1186/s12864-015-1593-2

**Published:** 2015-05-16

**Authors:** Prasoon Agarwal, Paul Collier, Markus Hsi-Yang Fritz, Vladimir Benes, Helena Jernberg Wiklund, Bengt Westermark, Umashankar Singh

**Affiliations:** Department of Immunology, Genetics and Pathology, Uppsala University, Science for Life Laboratory, Rudbeck Laboratory, Dag Hammarskjölds Väg 20, Uppsala, 75185 Sweden; EMBL, Core Facilities and Services, Meyerhofsstrasse 1, Heidelberg, D-69117 Germany

## Abstract

**Background:**

CGGBP1 is a repetitive DNA-binding transcription regulator with target sites at CpG-rich sequences such as CGG repeats and Alu-SINEs and L1-LINEs. The role of CGGBP1 as a possible mediator of CpG methylation however remains unknown. At CpG-rich sequences cytosine methylation is a major mechanism of transcriptional repression. Concordantly, gene-rich regions typically carry lower levels of CpG methylation than the repetitive elements. It is well known that at interspersed repeats Alu-SINEs and L1-LINEs high levels of CpG methylation constitute a transcriptional silencing and retrotransposon inactivating mechanism.

**Results:**

Here, we have studied genome-wide CpG methylation with or without CGGBP1-depletion. By high throughput sequencing of bisulfite-treated genomic DNA we have identified CGGBP1 to be a negative regulator of CpG methylation at repetitive DNA sequences. In addition, we have studied CpG methylation alterations on Alu and L1 retrotransposons in CGGBP1-depleted cells using a novel bisulfite-treatment and high throughput sequencing approach.

**Conclusions:**

The results clearly show that CGGBP1 is a possible bidirectional regulator of CpG methylation at Alus, and acts as a repressor of methylation at L1 retrotransposons.

**Electronic supplementary material:**

The online version of this article (doi:10.1186/s12864-015-1593-2) contains supplementary material, which is available to authorized users.

## Background

CGGBP1 is a DNA-binding, transcription regulatory protein shown to have binding sites on CGG tandem repeats as well as repetitive clusters of ribosomal RNA genes [1-3]. The CpG-richness of CGGBP1-binding sequences raises the question whether CpG methylation may be a mechanism underlying transcription-regulation by CGGBP1. Despite evidence of transcriptional silencing by binding of CGGBP1 to unmethylated CGG repeats [2,4], the effects of CGGBP1 on CpG methylation have never been studied. Recently CGGBP1-binding was demonstrated at repetitive DNA including transcription-regulatory regions of Alu-SINEs and L1-LINEs [5]. CGGBP1 acts as a growth-specific transcription suppressor of a subset of Alu-SINEs [5]. Unlike the gene-rich regions, the repetitive DNA e.g. peri-centromeric, sub-telomeric and satellite repeats as well as interspersed repeats, including Alu and LINE-1 elements carry high methylation levels [6-8].

Methylation of cytosine bases on DNA is a pivotal epigenetic mark important for development and differentiation [6,7,9-12] and importantly also required for suppression of transcription of repetitive elements in the genome [8]. Cytosine methylation has been most studied in the CpG context, although it also occurs in CHG and CHH contexts [13,14].

DNA methyltransferases either methylate cytosine bases *de novo* (DNMT3A and DNMT3B) [15] or at hemi-methylated sites during replication (DNMT1) [6,16,17], although context-specific *de novo* methylation by DNMT1 has been reported [12,18,19]. SUV39H, HDACs, HMTs, pRB, p23, DMAP1, PCNA and MBD2 are some proteins that regulate activities of DNMTs [6,7,17,20-24]. Of these, all except HDACs and pRB, are positive effectors of their activities and cytosine methylation. Erasure of CpG cytosine methylation involves oxidation and deamination of methylated cytosine by TET and AICDA proteins respectively followed by base-excision repair based on the complementary guanidine [25-27]. An interplay between positive and negative effectors of CpG methylation makes sure that even within the constitutive heterochromatin, cytosine methylation may not be 100% and an equilibrium between unmethylated and methylated cytosine bases is maintained. The factors restricting CpG methylation from invading all cytosine bases remains largely unknown. Unraveling the function of potential novel regulators of cytosine methylation such as CGGBP1 thus becomes important.

## Results

To elucidate the role of CGGBP1 in regulation of CpG methylation we performed global as well as targeted (at Alu and LINE-1 repeats) methylation analysis of genomic DNA from normal human fibroblasts after an acute depletion of CGGBP1.

**Figure 1:**
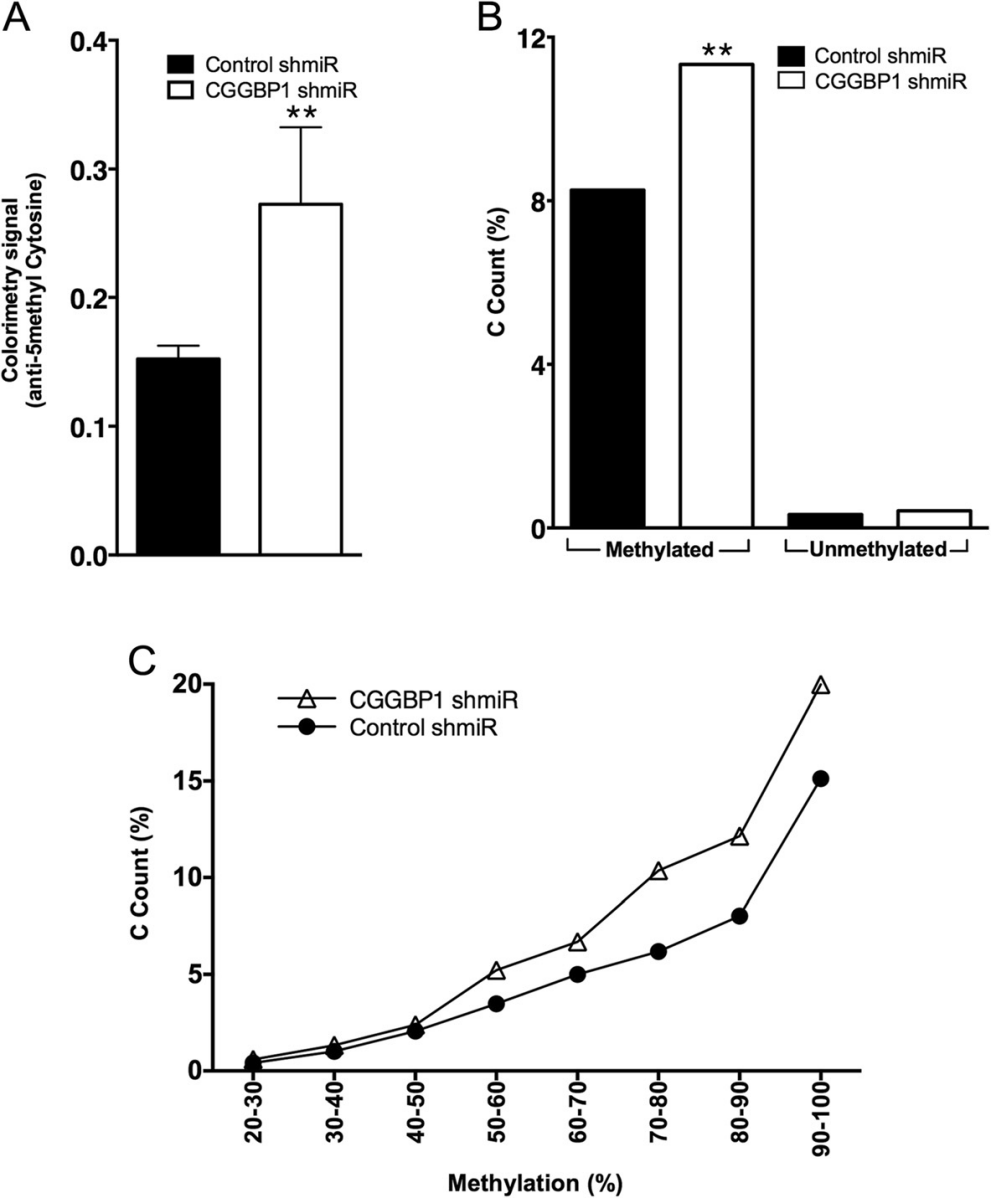
Global methylation changes upon CGGBP1-depletion. **A**: Colorimetric analysis reveals an increase in CpG methylation. Y-axis shows colorimetry signal from 3 independent assays (mean ± SEM). **B**: GeneSpring output showing changes in CpG methylation. The increase in methylation is significant between CGGBP1-depleted and Control samples. Y-axis shows C count (%, [calculated as C count x100/total number of nucleotides]). **C**: Frequency plotting of CpG methylation changes across different ranges of methylation. This plot shows binning of data depicted in 1B. X-axis shows methylation frequency bins and Y-axis shows C count (%).

1064Sk cells were transduced with control or CGGBP1-targeting shmiR-lentiviruses and CGGBP1-depletion was confirmed by western blotting (Additional file 1). Genomic DNA was extracted and used for methylation analysis by colorimetry using antibody directed against methyl-cytosine (Epigentek). The results showed that cytosine methylation was increased upon CGGBP1 depletion (Figure 1A; Ratio paired *T* test p=0.0211).

Then, paired-end sequencing was performed for the control and CGGBP1-depleted DNA after bisulfite treatment. The reads were mapped (only unique alignments reported) using Bismark [28] and the data further analyzed using GeneSpring v12.6.1 (Agilent). For bisulfite treatment, 100% unmethylated phage lambda DNA was used as spike-in control and bisulfite conversion efficiency in both samples was ascertained as 95% (full details in the section on methods). Since bisulfite treatment converts unmethylated cytosine bases to uracil (which eventually in PCR gives rise to adenine), an increase in cytosine content (C count; expressed as % of total bases sequenced) upon bisulfite treatment becomes a measure of methylated cytosine bases.

Supporting the colorimetry results, the bisulfite-treatment-sequencing experiment also showed increased cytosine content in CGGBP1-depleted cells in CpG context (Figure 1B). The ratio of methylated CpG to unmethylated CpG increased from 25.03 in control to 26.97 in CGGBP1 depleted sample (individual % methylated and % unmethylated C counts shown in Figure 1B). This showed that acute CGGBP1-depletion increased CpG methylation. The significance of increase in CpG methylation has been calculated using Fisher’s exact test (p < 0.01) inbuilt in GeneSpring.

A distribution of C% content with respect to Methylation% showed that this increase in CpG methylation upon CGGBP1-depletion was maximal at highly methylated regions exhibiting 70%–90% methylation (Figure 1C). This indicated that upon CGGBP1 depletion unmethylated regions did not become aberrantly methylated, rather already significantly methylated regions became slightly but significantly hypermethylated.

To ensure that (i) the small yet significant increases in methylation measured in terms of C count was not due to any base composition bias between the control and CGGBP1-depleted DNA samples, and (ii) that the changes in C count were indeed due to bisulfite conversion, we next performed Illumina paired-end sequencing without any bisulfite treatment on the same samples as used for sequencing after bisulfite conversion (mentioned above). The C count was found to be unaffected by CGGBP1-depletion in the absence of bisulfite conversion (Additional file 2 (A; black line versus green line)) but was significantly (Chi square test with Yate’s correction p < 0.0001) increased by CGGBP1-depletion in presence of bisulfite conversion (Additional file 2 (A; red line versus orange line)). Also, specifically in bisulfite-converted DNA, the relative changes in C counts (and G counts on reverse strand) were associated with inverse and significant changes in T counts (and A counts on reverse strand) when CGGBP1-shmiR samples were compared against Control shmiR with the latter values normalized to 1 (Fisher’s exact test p < 0.0001; Additional file 2 (B)). These results confirmed that the C count increase observed upon bisulfite-treatment-sequencing in CGGBP1-depleted sample was genuinely due to increased cytosine methylation.

Region annotation of cytosine bases exhibiting increased methylation showed that >99% of them were located in inter-genic regions (more than 5Kb from nearest known genes). The repetitive DNA sequences are silenced by methylation and CGGBP1 is a repeat-binding protein with affinity for unmethylated DNA. So we asked if the increase in methylation upon CGGBP1-depletion occurs at repetitive DNA. Non-overlapping sequences of −100 to +100 bps regions flanking the methylated cytosine bases were extracted and subjected to repeat identification by RepeatMasker [29]. A substantial fraction of the differentially methylated regions were located in repetitive regions of the genome as mentioned below: interspersed repeats (6.57%), small RNA (3.91%), simple repeats (21.69%) and satellite repeats (20.56%) (Additional file 3). CGGBP1 depletion thus seemed to increase cytosine methylation at repetitive DNA in the inter-genic regions.

Alu and LINE-1 repeats constitute a major fraction of interspersed repeats in our genome. They are silenced by CpG methylation and thus also serve as major cytosine methylation repositories. Also, Alu-SINEs and L1-LINEs have been recently shown to be major CGGBP1-binding sites [5]. To identify the changes in methylation occurring at Alu and LINE-1 repeats upon CGGBP1-depletion, we established PCR conditions to amplify Alu and LINE-1 repeats genome-wide from bisulfite-converted DNA (Additional file 4; and see methods for details). By this approach we could measure global changes in Alu and LINE-1 methylation on all cytosine residues in a 220 bp Alu amplicon and a 429 bp LINE-1 amplicon (benchmark lengths derived from distance between primers in the consensus sequences) (Additional file 4). The Alu and LINE-1 bisulfite PCR products were sequenced on PacBio platform and analyzed to reveal any changes in CpG methylation. The Alu PCR products showed some concatenation in sequencing library preparation but since the methylation change was being calculated as a drift from C to T, this did not affect the results. The mean ± S.D. lengths of the Control shmiR and CGGBP1 shmiR LINE-1 PCR products were heavily centered around the expected full-length amplicon (Additional file 5). Sequences for the entire length of amplicon for all fragments were not achieved, so we included in our analysis only sequences, which were at least 100 bases long. Out of >7000 Alu sequences per sample and >10000 LINE-1 sequences per sample, there were no duplicates, showing that the PCR product indeed amplified from different Alu and LINE-1 elements genome-wide. The alignment of the individual sequences against consensus Alu and LINE-1 sequences are shown in the files submitted to NCBI GEO database (GSE60784).

**Figure 2:**
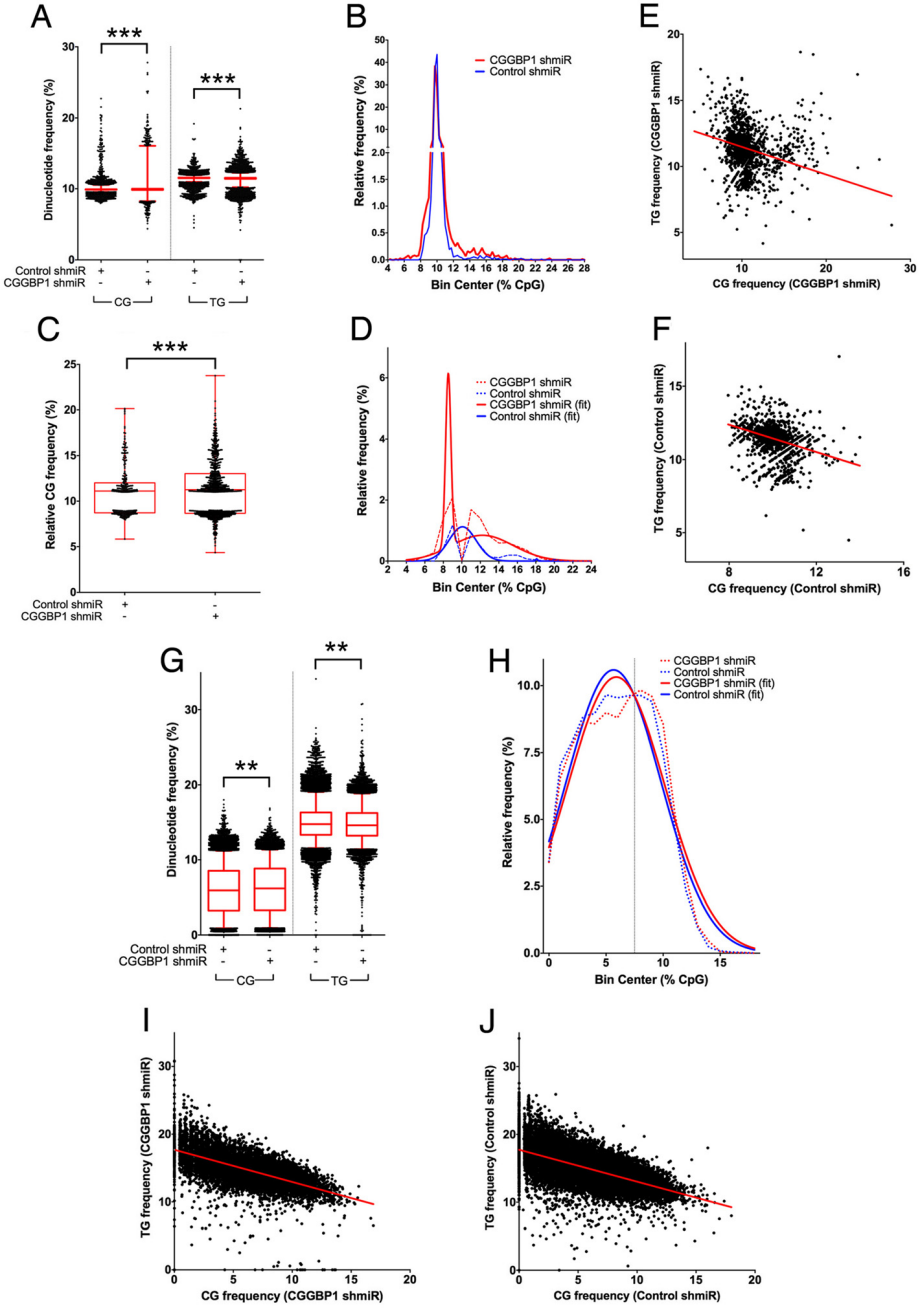
Alu and LINE-1 repeats exhibit methylation changes upon CGGBP1-depletion. **A**: Mean methylation increase on Alu repeats measured by CG frequency per PCR product sequence. Y-axis shows nucleotide frequency calculated per sequence. X-axis shows the samples and treatments. **B**: Frequency distribution of Alu methylation across different ranges shows decrease (<8%) and increase (>12%) at extremes in CGGBP1 shmiR sample as compared to Control shmiR sample. Y-axis shows relative frequencies of CG (a measure of methylation; normalized for different number of sequences per sample). **C**: Plotting of the >12% and <8% methylation subset from **B** shows the tailing of differentially methylated sequences at both extremes clearly. **D**: Frequency plot of data plotted in **C** and best curve fit shows sum-of-two-Gaussian fit for CGGBP1-depleted sample and a single Gaussian distribution for control sample suggesting that indeed there are two groups of methylation levels for Alus in CGGBP1 shmiR but only one group of methylation level in Control shmiR sample. **E** and **F**: Increase in CpG content negatively correlated with TpG frequency in both samples establishing the fact that the changes in cytosine content was indeed due to bisulfite conversion of unmethylated cytosines. **G**: Increase in methylation on LINE-1 elements was significant with no bidirectional heterogeneity as seen for the Alus. **H**: Frequency plotting showed that <7.5% (marked with dotted line) methylation was prevalent in control sample, but >7.5% methylation was prevalent in CGGBP1-depleted sample (values normalized for different number of sequences per sample). **I** and **J**: CpG and TpG frequencies on LINE-1 exhibited inverse correlations in CGGBP1 and control shmiR samples establishing the fact that the changes in cytosine content was indeed due to bisulfite conversion of unmethylated cytosines.

The level of methylation of CpG dinucleotides in this case was measured as the frequency of CpG. The mean CpG methylation on Alus was significantly increased (Figure 2A; CpG increased and TpG decreased; *T* test p < 0.0001). Although there was an increase in Alu methylation overall, an inspection of the distribution of methylation frequencies indicated two different directions of methylation change. A major fraction had increased (>12%) methylation and a minor fraction had decreased (<8%) methylation (Figure 2B) In the CGGBP1 shmiR sample, the deviation of <8% and >12% fractions from expected (distribution of <8% and >12% fractions in Control shmiR sample) was highly significant (Chi square test, p < 0.001). Plotting only the sequences having CpG methylation <8% and >12% further highlighted the possible bidirectional change in Alu methylation (Figure 2C). A frequency plot of Alu methylation <8% and >12% subjected to best distribution fit identified a sum-of-two-Gaussians clearly in CGGBP1-depleted sample but only a major single Gaussian distribution in Control sample (Figure 2D). That the changes in CpG content indeed occurred due to bisulfite treatment was confirmed because the changes in CpG content correlated inversely with changes in TpG content as bisulfite treatment causes a C- > T drift (Figures 2E and F) (for both correlation plots, R^2^ > 0.18 with p < 0.0001).

CpG methylation on LINE-1 also showed a significant increase after CGGBP1-depletion (Figure 2G; CpG increase and TpG decrease, *T* test p < 0.001). The control samples had higher content of <7.5% methylated LINE-1 elements, whereas the population of >7.5% methylated LINE-1 repeats increased in CGGBP1-depleted sample (Figure 2H) (distribution shift significant; Chi square test, p < 0.001). By plotting the CpG content against TpG content on the LINE-1 repeats, again we found a strong inverse correlation, confirming that the increase in CpG content indeed occurs due to methylation (Figures 2I and J) (R^2^ > 0.4 and p < 0.0001). Since a fraction of the TpG content is a part of the native sequence of Alu and LINE-1 elements which does not arise out of C- > T conversion, the observed inverse correlation between the CpG and TpG may actually underrepresent the true inverse correlation between methylated CpG versus bisulfite conversion-derived TpG.

## Discussion

To unravel the possibility that CGGBP1 with target sites at CpG-rich sequences may also function as a possible mediator of CpG methylation we performed global and targeted methylation analysis of genomic DNA from normal human fibroblasts after an acute depletion of CGGBP1. The results from these experiments now clearly show that CGGBP1 is a net negative regulator of CpG methylation. The regions most affected by CGGBP1-dependent methylation regulation are inter-genic regions including simple repeats, satellite DNA and interspersed repeats. An in-depth nucleotide level analysis of the methylation changes at discrete CpG nucleotides will generate more information about the DNA sequence context in which CGGBP1 targets methylation at CpG sites. Although CGG repeats constitute a major binding site for CGGBP1, there are CGG-free regions to which it binds. These include CDKN1A promoter [30], telomeric repeats [31], a CGG repeat-free SNP rs11597367*G [32] and Alu and LINE promoters in addition to other repetitive elements in the genome [5]. Thus the effect of CGGBP1 depletion on methylation at CGG repeat-free regions is not surprising.

Targeted analysis of methylation on Alu and LINE-1 repeats genome-wide upon CGGBP1-depletion and demonstrated an increase in CpG methylation. These regions are usually constitutively inactivated by, and carry a major fraction of, CpG methylation. Obviously these regions are under constant influence of mechanisms that ensure high levels of methylation. This would include constant surveillance by DNA methyltransferases as well as constitutive heterochromatin-associated histone modifiers. Since cytosine methylation erasure mechanisms, including oxidation, deamination and subsequent base excision processes, would counteract methylation at these repetitive regions, they may not be 100% methylated at any particular time point. The mechanism underlying methylation increase upon CGGBP1-depletion may either involve an increase of *de novo* methylation, or a decrease of methylation erasure, or both combined. Given the already high methylation levels at repetitive regions, a further augmentation of *de novo* methyltransferases activity at these elements is difficult to comprehend. Therefore the possibility that CGGBP1 depletion mitigates methylation erasure appears more likely. An analysis of the recently published gene expression profile of CGGBP1-depleted cells supports our current findings. From this dataset, an identification of expression profiles of genes with known functions in cytosine methylation regulation showed that CGGBP1-depletion decreases the expression of genes involved in cytosine/methyl-cytosine oxidation (the APOBEC family of enzymes) and increases the expression of positive effectors of CpG methylation including DNMT1 (Additional file 6). Alternatively, since CGGBP1 has affinity for unmethylated repetitive DNA [4] it is possible that CGGBP1 shields sub-regions of repetitive DNA from *de novo* methyltransferases activity. Down regulation of CGGBP1 will then expose these regions to methylation-incorporating machinery. This would result in methylation increase, as observed in the present investigation.

Interestingly, even if CGGBP1 depletion increases cytosine methylation, it does so not at unmethylated regions or regions with very low cytosine methylation (for example CpG islands associated with gene-rich regions), rather at already significantly methylated repetitive DNA regions. A decrease in methylation at repetitive DNA or an increase in methylation at CpG islands could be high in magnitude, but a further increase in methylation at already highly methylated regions can only be limited. This may explain the only low but highly significant changes in methylation levels observed upon CGGBP1 depletion.

The possibility of bidirectional change in Alu CpG methylation suggests that different Alu elements may be subjected to different mechanisms of CpG methylation regulation by CGGBP1. Further work is needed to identify which kinds of Alu elements exhibit decreased methylation upon CGGBP1-depletion. A classification of Alu sequences obtained in the current work into different sub-families is not possible due to two factors: (i) generation of chimeric Alu sequences in PCR due to annealing and cross-amplification of highly similar Alu sequences belonging to different subfamilies, and (ii) site-specific C- > T variations could not be identified as differences in original genomic DNA or as occurring due to bisulfite conversion. Although the primers were intended to be selected from as highly conserved regions of Alus as possible, it is important to note that due to sequence variations between different Alu elements, there could be a minor PCR amplification bias against the Alu elements which do not match completely with the primer sequences or have unmethylated CpG dinucleotides in the priming region.

The approach employed in this study to study genome-wide methylation changes upon CGGBP1 depletion measures the overall changes in cytosine methylation without giving a microscopic base-level information about the methylation targets of CGGBP1. These data give a sound platform to build upon to uncover the sequence contexts in which CGGBP1 exerts methylation regulation at specific sites.

Though several positive effectors of DNA methylation are known, to the best of our knowledge, apart from histone-modifying proteins HDACs and HMTs and pRB, this is the first factor described to have negative effects on cytosine methylation. We therefore have discovered a unique feature of CGGBP1 that is important for regulation of DNA methylation. This has implications on silencing of Alu and LINE-1 repeats, heterochromatin formation on simple and satellite repeats and hence on genome integrity and function.

## Conclusions

CGGBP1 depletion results in increased CpG methylation at repetitive DNA sequences. These include Alu-SINEs and L1-LINEs. A subset of Alu-SINEs however display decreased methylation suggesting a bi-directionality in the effects of CGGBP1 on CpG methylation on Alu-SINEs. Gene expression data suggest that a transcriptional deregulation of CpG methylation-regulatory genes could underlie this effect of CGGBP1.

## Methods

### CpG methylation measurement by colorimetry

CpG methylation in CGGBP1 shmiR or Control shmiR-transduced cells was measured by using MethylFlash Methylated DNA Quantification Kit (Colorimetric) from EpiGentek. Signals were extracted using colorimeter (Cole Parmer). Raw signals from 3 colorimetric analyses were plotted.

### Cell culture and shmiR transduction

1064Sk normal human foreskin fibroblasts (passage 12) were cultured in MEM (SIGMA), 10% FCS (SIGMA) and 0.05% Glutamine (SIGMA). Passaging was done using Trypsin (SIGMA). Lentiviral shmiR against CGGBP1 (cocktail of three target sequences) or control (non-targeting) were obtained from ThermoScientific. Transductions were done at an MoI of 6 using Polybrene. After 24 h and 72 h of transduction, medium was changed. At 96 h post-transduction, cells were harvested and CGGBP1 knockdown confirmed by western blot (Additional file 1) as described before. Genomic DNA was extracted from same samples using Qiagen Genomic DNA extraction kit.

### Bisulfite conversion of DNA and lIlumina library preparation

Bisulfite conversion of DNA was performed using EZ methylation Kit (Zymo Research). Library prep was carried out using the NEXTflex Bisulfite (cat# 5119–01) Sequencing kit provided by Bioo Scientific, according to the provided protocol. Volumes were calculated for the two samples that gave 1ug input into the fragmentation and combined with nuclease free water to 130ul in a Covaris tube and fragmented using our established protocol (we used the Covaris S2 acoustic sonicator paired with Covaris MicroTube AFA Fibre pre-split (520045) tubes with the following settings: Duration: 50 sec, Duty Cycle: 20%, Intensity: 5.0, Cycles/Burst: 200). Fragmented DNA was then speed-vac concentrated to 39ul working volume for the library prep reaction and combined with 1ul of 500 pg/ul Lambda DNA spike.

### Illumina sequencing and data analysis

Samples were run on Illumina Sequencers by standard methods. In addition to the library made from bisulfite-treated DNA, libraries prepared from untreated DNA samples were also sequenced. Paired-end reads were aligned using Bowtie 2 [33] for non-bisulfite-treated samples or Bowtie 2 in Bismark for bisulfite-treated samples. The sequences were aligned against a joint human + lambda reference.

The under-conversion rate was derived by subsetting the lambda read alignments and dividing the number of C-C matches with the total coverage of C positions in the lambda genome (lambda DNA was 100% unmethylated, so all remaining Cs were ascribed as occurring due to under-conversion). In both samples the Lambda DNA spike in controls indicated 95% conversion efficiency. The nucleotide content of non-bisulfite-treated samples was calculated using Compseq [34]. For bisulfite-treated samples, the Bismark output (BAM files) was used as input in GeneSpring (Agilent) and subjected to derivation differentially methylated cytosine residues and their annotation with respect to presence in or vicinity of known genes. The parameters used in GeneSpring were as follows: Methylation identification using Fisher’s test p = 0.01, bisulfite conversion error rate=0.05 (5%) using external standard (lambda DNA in this case). The flanking regions of differentially methylated cytosine residues were pulled from Hg19 assembly using tools embedded in Galaxy Project [35]. The repeat content of the sequences were analyzed using RepeatMasker [29].

### Alu and LINE-1 amplification, PacBio sequencing and data analysis

Alu and LINE-1 repeats were amplified genome-wide from bisulfite-converted DNA samples using the following primers: GAGGTCGAGGCGGGAGGATCG and CGTTTAGGTTGGAGTGTAGTGGCGCG for Alu repeats (amplicon size 220 bps from Alu consensus sequence), and ATTTTTGTATTTTTATTTGAGGTAT and AACTATAATAAACTCCACCCAATTC for LINE-1 repeats (amplicon size 429 bps from LINE-1 consensus sequence).

The consensus amplicon sequences are the following:

Alus:

GAGGCCGAGGCGGGAGGATCGCTTGAGCCCAGGAGTTCGAGACCAGCCTGGGCAACATAGCGAGACCCCGTCTCTACAAAAAATACAAAAATTAGCCGGGCGTGGTGGCGCGCGCCTGTAGTCCCAGCTACTCGGGAGGCTGAGGCAGGAGGATCGCTTGAGCCCAGGAGTTCGAGGCTGCAGTGAGCTATGATCGCGCCACTGCACTCCAGCCTGGGC.

LINE-1:

AACTATAATAAACTCCACCCAATTCAAACTTCCGACCACTTTATTTACCGACTCAAACCTAACAATAACTAACACGTCCCCTCCCCCAACCTCACTACCGCCTTACAATTTAATCTCAAACTACTACGCTAACAATAAATAAAACTCTAATAAACATAAAACCCTCTCAAACTAAACGCGACATATAATCTCCTAATATATCATTTACTAATCGCATTAAAAAAACACAATATTAAAATAAAAATAACCCAATTTTCCAAATACCATCTATCACCCCTTTTCTTTAACTAAAAAAAAAAATTCCCTAACCCCTTACACTTCCGTACTAAACATACTCGTACTTCACTCACATACACCCACTATCCTTCACCCACTATCTAACACTTCCCATTAAAATAAACCTAATACCTCAAATAAAAATACAAAAAT

PCR cycling conditions were: 35 cycles of 94°C 30 sec, 54°C 40 sec and 72°C 1 min for LINE-1 and 94°C 5 min, 20 cycles of 94°C 30 sec, 50°C 25 sec and 72°C 45 sec for Alus. Several reactions for Alus were pooled in because of low yield. Increasing the number of cycles gave concatenated products due to an imperfect duplicated repeat-nature of Alu amplicon. The approximately 90 bps product seen in Alu PCR product is due to genuine annealing of the forward primer in Alu right arm. The 220 bps Alu product is derived from forward primer annealing in left arm. A quality control gel picture of Alu and LINE-1 PCR products is shown in Additional file 2. The indicated 220 and 429 bps bands were excised out using Qiagen gel elution kit and subjected to PacBio Library preparation and sequencing. SMRT bell libraries were produced using Pacific Biosciences 1.0 template prep kit according to the manufacturer’s instructions. Libraries were constructed following Pacific Biosciences 500 bp template preparation and sequencing protocol. Following SMRT bell construction, v2 primers were ligated and P5 polymerase was bound to the SMRT bell library. Using C3 chemistry, the polymerase-template complexes were sequenced on the Pacific Biosciences *RS II* real-time sequencer. Each amplicon SMRT bell library was sequenced on one SMRT cell using a 180 min movie time. Using Pacific biosciences SMRT analysis software 2.2, sequencing reads were filtered by quality and length and single-molecule consensus reads generated from the insert template. Reads obtained were matched against target consensus sequences using *Supermatcher* (EMBOSS) [36].

### Statistics

Colorimetry data were analysed by performing *T* test in MS Excel.

Chi square/Fisher’s Exact tests for significance of CpG content variation was performed sing statistical options embedded in GeneSpring (Agilent).

T tests for PacBio sequencing data, correlation coefficients and p values of CG to TG content changes were calculated using GraphPad Prism. Nucleotide content changes in non-bisulfite treated Illumina sequencing data were done using MS Excel and GraphPad Prism. All graphs were generated using GraphPad Prism.

## Additional files

Additional file 1:
**Western blot analysis of CGGBP1 depletion: The CGGBP1 band corresponds to 20 KDa and shows a clear decrease in CGGBP1 shmiR sample as compared to Control shmiR sample.** ACTB (beta-actin) was used as a loading control. Additional file 2:
**Comparison with unconverted DNA showed specificity of effect of bisulfite-conversion on C% content changes.** **A**: Sequencing of non-bisulfite-treated samples showed no significant changes in C content. Y-axis shows nucleotide count (%), which was split into C or non-C fractions as plotted on X-axis. The corresponding C or non-C values from CGGBP1 shmiR or Control shmiR samples were subjected to Chi-square test. **B**: A plot of relative change (CGGBP1 shmiR/Control shmiR) in individual nucleotide frequencies in bisulfite-converted or non-converted DNA samples; Y axis shows the nucleotide frequency in CGGBP1-shmiR divided by Control shmiR, thus normalising all control shmiR values to 1. The treatment of bisulfite and the nucleotides are specified in legend and X-axis respectively. The change in relative frequencies of C and G versus T and A respectively across bisulfite treatment groups is significant as determined using a Chi-square test (indicated by asterisks). Additional file 3:
**Pie chart showing repeat-content identification in the 100 bps flanking regions of differentially methylated cytosines between CGGBP1-shmiR and Control shmiR samples.** More than 99% of the differentially methylated cytosines were located >5Kb away from the nearest genes. Additional file 4:
**Quality control gel picture of Alu and LINE-1 PCR from bisulfite-converted genomic DNA: The Alu primers amplify a 220 bps product and LINE-1 primers amplify a 429 bps product (size calculated based on consensus sequences, though the amplicon is expected to be a mix of different fragments with minor differences in molecular weight).** The non-converted DNA did not give rise to amplifications for both primer sets. The indicated fragments were eluted from the gel and subjected to sequencing. Additional file 5:
**Size distribution of sequence reads for LINE-1 PCR products from bisulfite-treated DNA:** **A**: **Distribution of read lengths in bins of 10 bases each from 100 bases onwards shows that the maximum number of reads are of the size range between 400 and 500 bps.** **B**: The raw lengths of all the reads included in the analysis for the two samples have been shown. The remarkable accumulation of reads in the expected sub-500 bps region is clearly visible. The mean sequence sizes for Control shmiR and CGGBP1 shmiR LINE-1 PCR products are 394.3 ± 75.27 and 401.0 ± 69.01 bps respectively. Additional file 6:
**Analysis of effects of CGGBP1-depletion on expression of CpG methylation-affecting genes from a previous study (Agarwal et al.,** ***Cell Cycle***, **2014).** **A**: A heat map of selected set of genes known to be involved in cytosine methylation regulation. The Control- and CGGBP1-shmiR datasets are each represented by three replicates. **B**: A quantification of the expression values shown in heat map (A) presented as mean + SEM. *T* test has been employed to mark out the significantly varying genes (asterisk marked). *=p < 0.01, **=p < 0.001, ***=p < 0.0001.
