# Supplementary figures and images for: CGGBP1 mitigates cytosine methylation at repetitive DNA sequences
Source: BMC Genomics. 2015 May 16;16(1):390. doi: 10.1186/s12864-015-1593-2 (PMC4432828; doi:10.1186/s12864-015-1593-2)

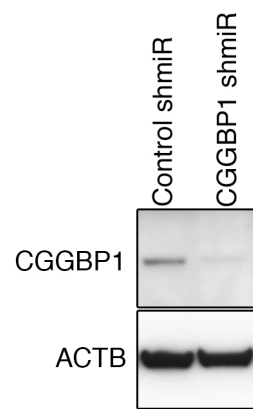

Supplement: Additional file 1: — Western blot analysis of CGGBP1 depletion: The CGGBP1 band corresponds to 20 KDa and shows a clear decrease in CGGBP1 shmiR sample as compared to Control shmiR sample. ACTB (beta-actin) was used as a loading control. [file 12864_2015_1593_MOESM1_ESM.pdf]

**A**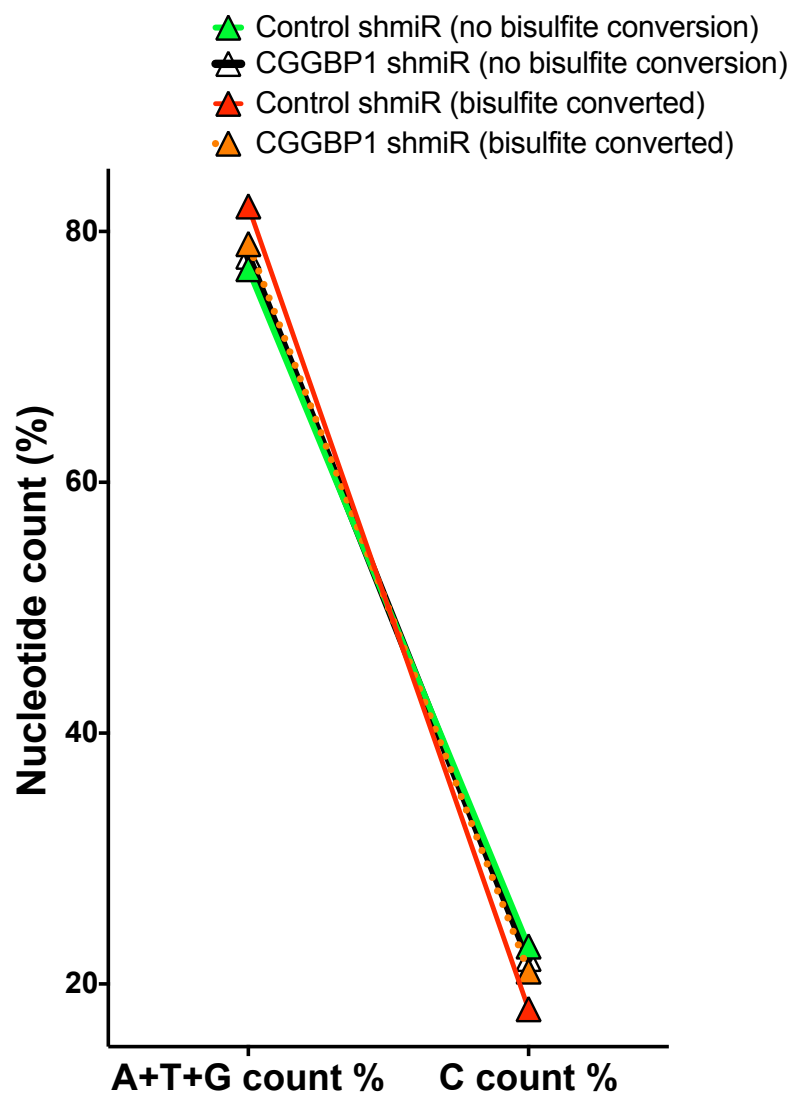**B**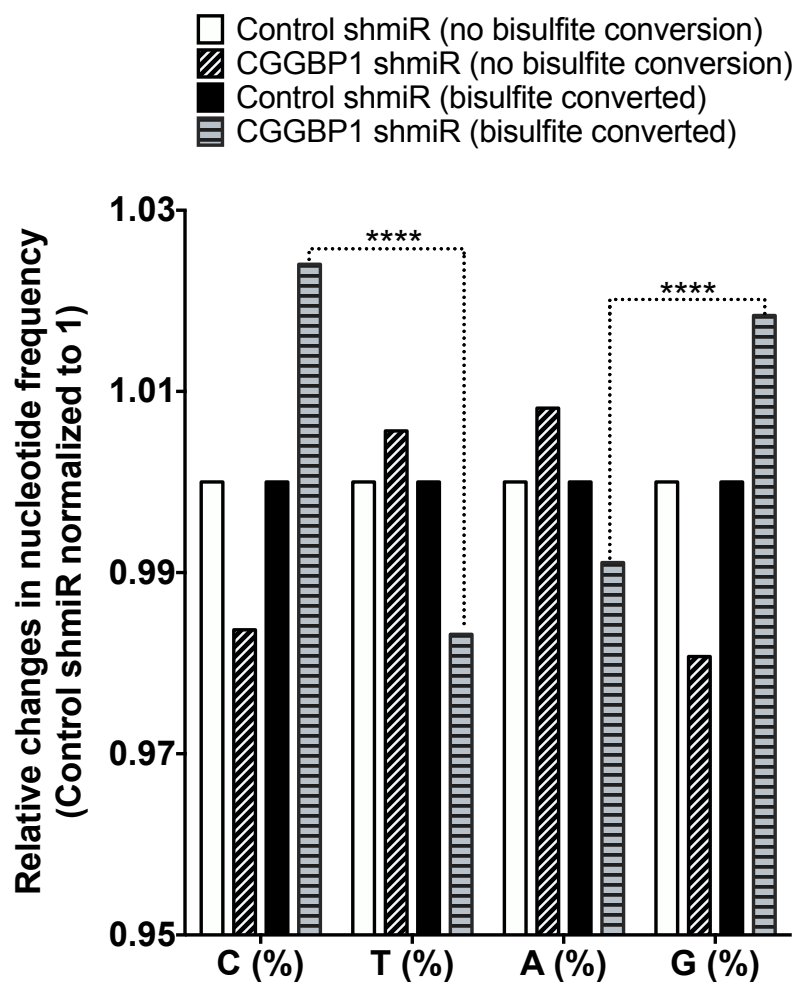

Supplement: Additional file 2: — Comparison with unconverted DNA showed specificity of effect of bisulfite-conversion on C% content changes. A: Sequencing of non-bisulfite-treated samples showed no significant changes in C content. Y-axis shows nucleotide count (%), which was split into C or non-C fractions as plotted on X-axis. The corresponding C or non-C values from CGGBP1 shmiR or Control shmiR samples were subjected to Chi-square test. B: A plot of relative change (CGGBP1 shmiR/Control shmiR) in individual nucleotide frequencies in bisulfite-converted or non-converted DNA samples; Y axis shows the nucleotide frequency in CGGBP1-shmiR divided by Control shmiR, thus normalising all control shmiR values to 1. The treatment of bisulfite and the nucleotides are specified in legend and X-axis respectively. The change in relative frequencies of C and G versus T and A respectively across bisulfite treatment groups is significant as determined using a Chi-square test (indicated by asterisks). [file 12864_2015_1593_MOESM2_ESM.pdf]

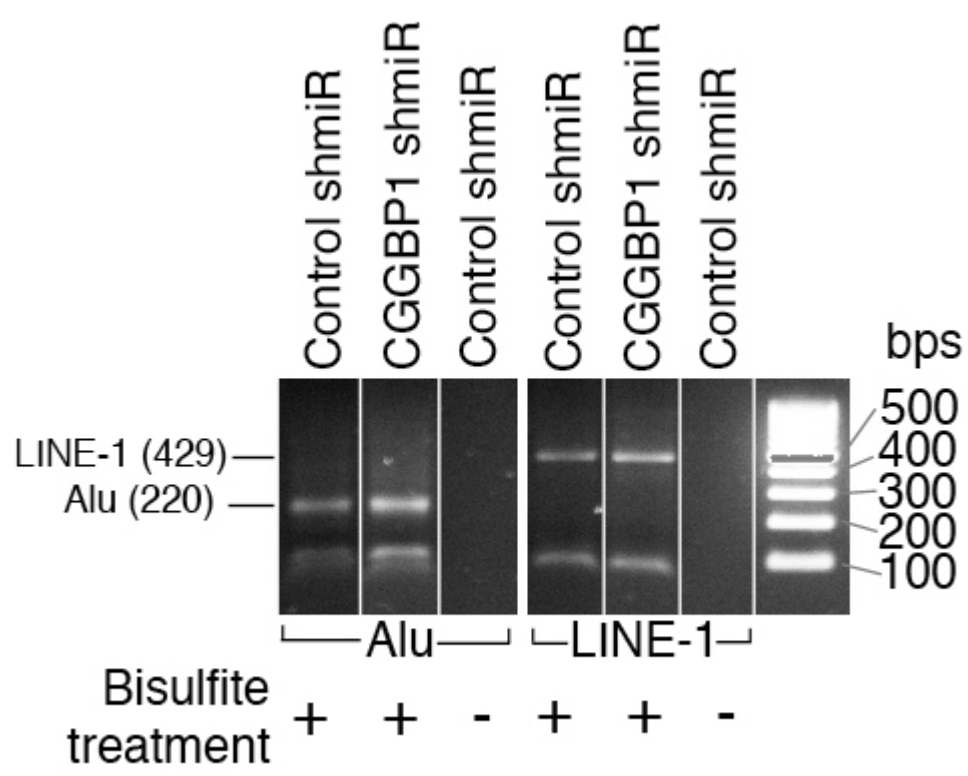

Supplement: Additional file 4: — Quality control gel picture of Alu and LINE-1 PCR from bisulfite-converted genomic DNA: The Alu primers amplify a 220 bps product and LINE-1 primers amplify a 429 bps product (size calculated based on consensus sequences, though the amplicon is expected to be a mix of different fragments with minor differences in molecular weight). The non-converted DNA did not give rise to amplifications for both primer sets. The indicated fragments were eluted from the gel and subjected to sequencing. [file 12864_2015_1593_MOESM4_ESM.pdf]

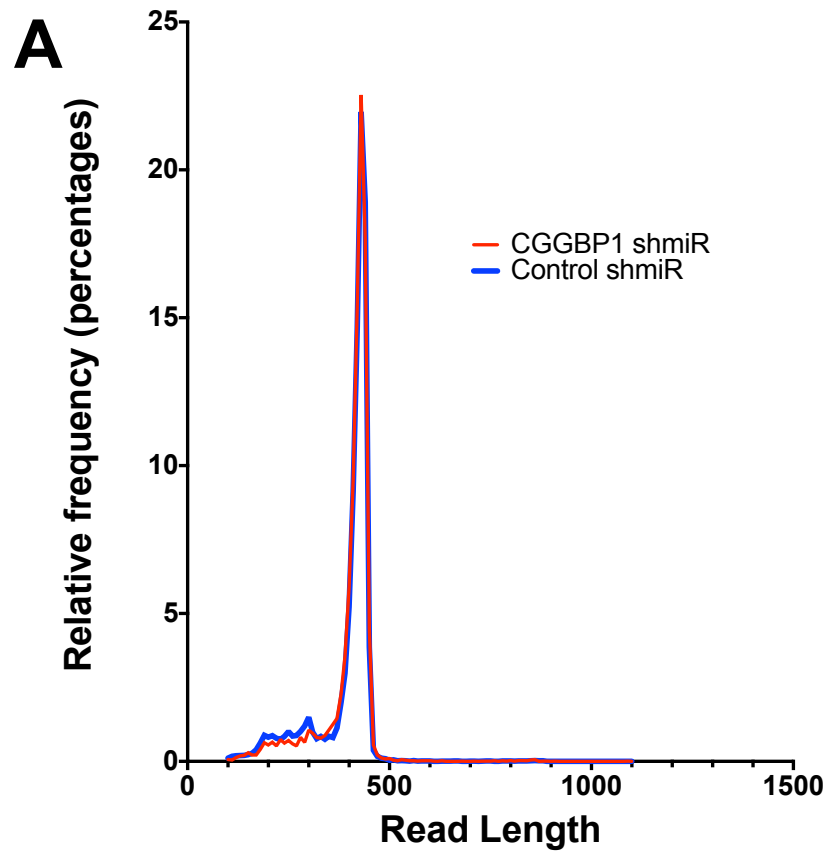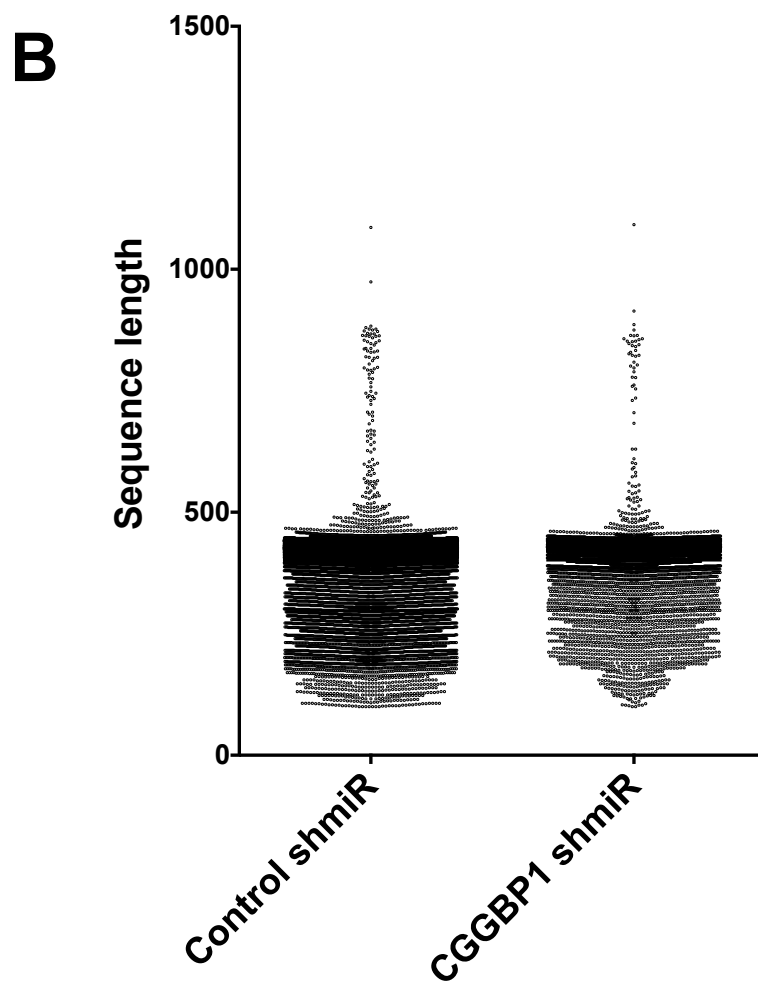

Supplement: Additional file 5: — Size distribution of sequence reads for LINE-1 PCR products from bisulfite-treated DNA: A: Distribution of read lengths in bins of 10 bases each from 100 bases onwards shows that the maximum number of reads are of the size range between 400 and 500 bps. B: The raw lengths of all the reads included in the analysis for the two samples have been shown. The remarkable accumulation of reads in the expected sub-500 bps region is clearly visible. The mean sequence sizes for Control shmiR and CGGBP1 shmiR LINE-1 PCR products are 394.3 ± 75.27 and 401.0 ± 69.01 bps respectively. [file 12864_2015_1593_MOESM5_ESM.pdf]

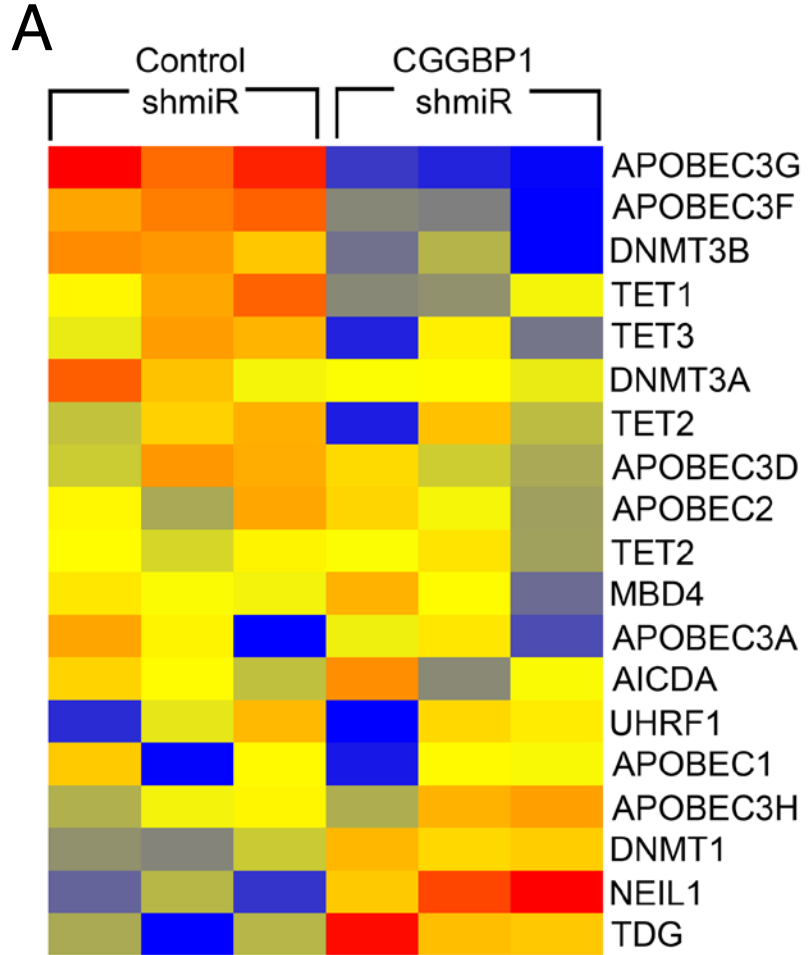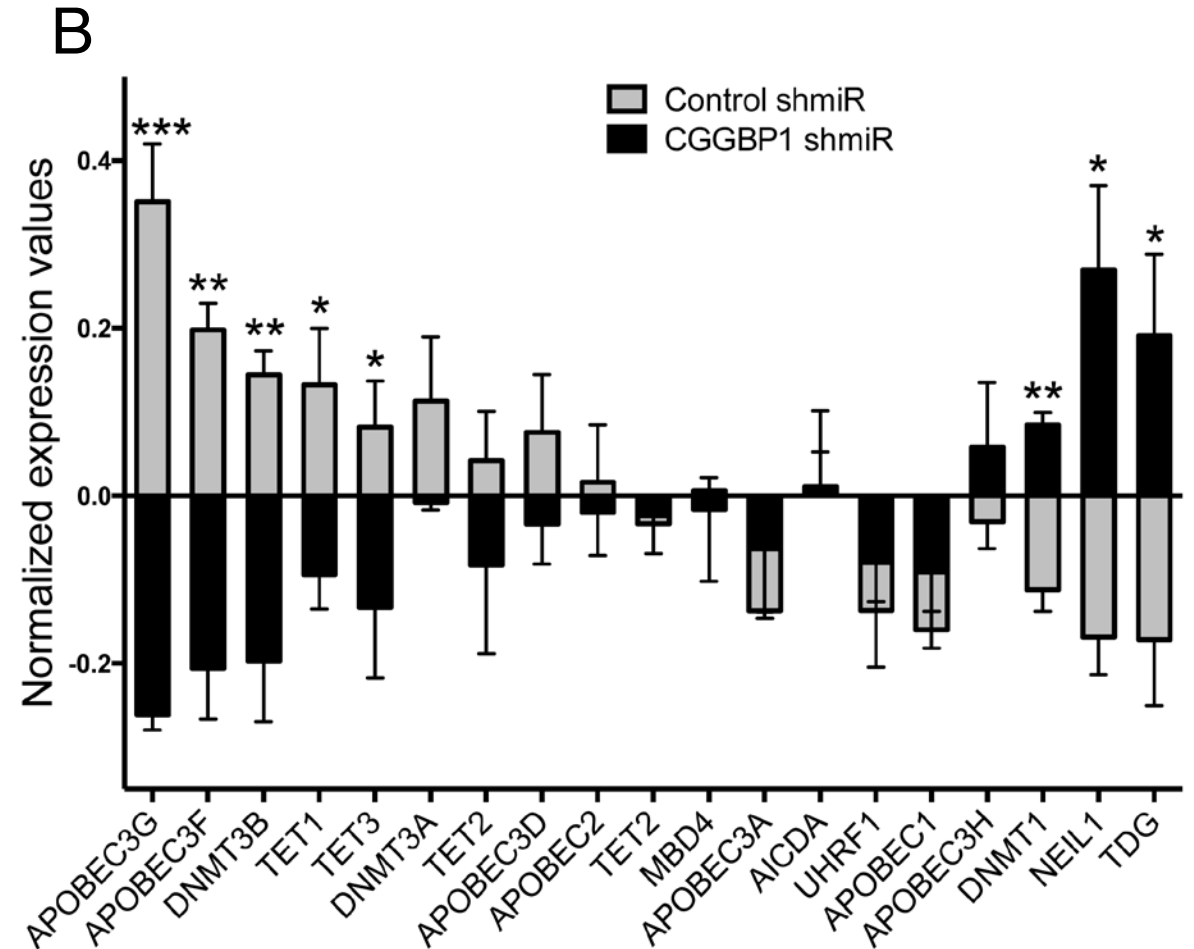

Supplement: Additional file 6: — Analysis of effects of CGGBP1-depletion on expression of CpG methylation-affecting genes from a previous study (Agarwal et al., Cell Cycle, 2014). A: A heat map of selected set of genes known to be involved in cytosine methylation regulation. The Control- and CGGBP1-shmiR datasets are each represented by three replicates. B: A quantification of the expression values shown in heat map (A) presented as mean + SEM. T test has been employed to mark out the significantly varying genes (asterisk marked). *=p < 0.01, **=p < 0.001, ***=p < 0.0001. [file 12864_2015_1593_MOESM6_ESM.pdf]
