# Supplementary material for: CGGBP1 mitigates cytosine methylation at repetitive DNA sequences
Source: BMC Genomics. 2015 May 16;16(1):390. doi: 10.1186/s12864-015-1593-2 (PMC4432828; doi:10.1186/s12864-015-1593-2)
Supplement: Additional file 3: — Pie chart showing repeat-content identification in the 100 bps flanking regions of differentially methylated cytosines between CGGBP1-shmiR and Control shmiR samples. More than 99% of the differentially methylated cytosines were located >5Kb away from the nearest genes. [file 12864_2015_1593_MOESM3_ESM.pdf]

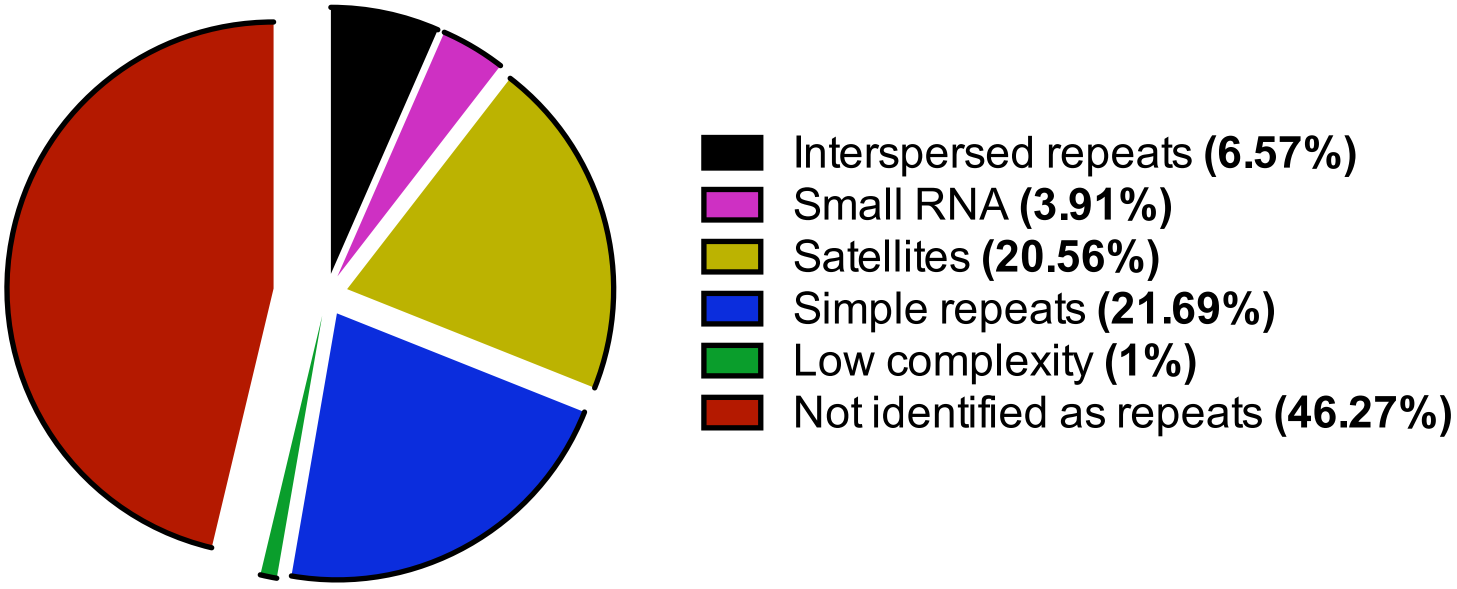
Interspersed repeats (6.57%)
Small RNA (3.91%)
Satellites (20.56%)
Simple repeats (21.69%)
Low complexity (1%)
Not identified as repeats (46.27%)
